# Supplementary material for: MetaRibo-Seq measures translation in microbiomes
Source: Nat Commun. 2020 Jun 29;11:3268. doi: 10.1038/s41467-020-17081-z (PMC7324362; doi:10.1038/s41467-020-17081-z)
Supplement: Supplementary file 10 — Supplementary Data 7 [file 41467_2020_17081_MOESM10_ESM.zip › File2/Confidence_High_Taxonomy/200881_out.krona.html]

Javascript must be enabled to view this page.

members
magnitude
magnitudeUnassigned
count
unassigned
taxon
rank

200881\_out

153

153
2
superkingdom

153
1239
phylum

186801
class
153

153
order
186802

186803
family
147

1
genus
830

1

SRS063190\_contig\_number\_4505
species
45851

genus
841
2

1

SRS015579\_contig\_number\_20423
species
2049040


SRS043001\_contig\_number\_10984
species
166486
1


SRS011302\_contig\_number\_30981SRS012969\_contig\_number\_25264SRS013158\_contig\_number\_contig-100\_154.42660SRS013521\_contig\_number\_8507SRS013951\_contig\_number\_28935SRS014235\_contig\_number\_45103SRS014313\_contig\_number\_contig-100\_5038.40720SRS014613\_contig\_number\_5042SRS014736\_contig\_number\_1419SRS014923\_contig\_number\_20449SRS014948\_contig\_number\_24343SRS015065\_contig\_number\_contig-100\_3759.45255SRS015190\_contig\_number\_16524SRS015217\_contig\_number\_8029SRS015486\_contig\_number\_contig-100\_203.25594SRS015794\_contig\_number\_12527SRS016018\_contig\_number\_14758SRS016203\_contig\_number\_contig-100\_1719.84072SRS016897\_contig\_number\_contig-100\_15326.15327SRS017103\_contig\_number\_contig-100\_6578.237449SRS017191\_contig\_number\_10453SRS017433\_contig\_number\_17883SRS018427\_contig\_number\_contig-100\_562.186726SRS019161\_contig\_number\_contig-100\_3539.236409SRS019178\_contig\_number\_contig-100\_17821.17822SRS019267\_contig\_number\_contig-100\_307.63530SRS019601\_contig\_number\_2273SRS019685\_contig\_number\_contig-100\_816.231700SRS020233\_contig\_number\_60136SRS021484\_contig\_number\_contig-100\_318.177937SRS022137\_contig\_number\_21385SRS022524\_contig\_number\_16306SRS022713\_contig\_number\_3023SRS023346\_contig\_number\_contig-100\_501.60626SRS023526\_contig\_number\_22936SRS024435\_contig\_number\_45564SRS024492\_contig\_number\_7863SRS042628\_contig\_number\_29840SRS043411\_contig\_number\_17578SRS043667\_contig\_number\_7029SRS043701\_contig\_number\_16135SRS043768\_contig\_number\_32912SRS044535\_contig\_number\_20451SRS045244\_contig\_number\_contig-100\_41.71105SRS045826\_contig\_number\_20929SRS046717\_contig\_number\_contig-100\_3343.89188SRS047044\_contig\_number\_contig-100\_135.101459SRS047433\_contig\_number\_20225SRS048060\_contig\_number\_4878SRS048164\_contig\_number\_32370SRS048870\_contig\_number\_contig-100\_1023.140141SRS048870\_contig\_number\_contig-100\_2101.141219SRS049900\_contig\_number\_contig-100\_2078.123096SRS049959\_contig\_number\_24372SRS050026\_contig\_number\_11311SRS050422\_contig\_number\_19935SRS050941\_contig\_number\_3516SRS051610\_contig\_number\_9706SRS051882\_contig\_number\_19838SRS052697\_contig\_number\_contig-100\_8469.204849SRS053214\_contig\_number\_22883SRS053335\_contig\_number\_contig-100\_202.104681SRS054590\_contig\_number\_contig-100\_203.29212SRS055966\_contig\_number\_contig-100\_6514.6515SRS055982\_contig\_number\_18282SRS057478\_contig\_number\_9019SRS057480\_contig\_number\_contig-100\_23232.23233SRS058723\_contig\_number\_contig-100\_701.63936SRS063370\_contig\_number\_13393SRS063985\_contig\_number\_32458SRS064276\_contig\_number\_19616SRS074586\_contig\_number\_953SRS074964\_contig\_number\_contig-100\_31.60745SRS075021\_contig\_number\_contig-100\_1396.61993SRS075716\_contig\_number\_2281SRS075773\_contig\_number\_33969SRS076804\_contig\_number\_12507SRS076976\_contig\_number\_contig-100\_13031.13032SRS077194\_contig\_number\_contig-100\_1040.193060SRS077231\_contig\_number\_contig-100\_2225.157765SRS077294\_contig\_number\_19464SRS077335\_contig\_number\_contig-100\_2675.58846SRS078176\_contig\_number\_23945SRS078665\_contig\_number\_contig-100\_6599.61154SRS097920\_contig\_number\_32149SRS097958\_contig\_number\_25263SRS098073\_contig\_number\_contig-100\_4156.50146SRS098073\_contig\_number\_contig-100\_11075.87243SRS098644\_contig\_number\_contig-100\_830.280267SRS100907\_contig\_number\_contig-100\_37004.37005SRS104084\_contig\_number\_contig-100\_548.90874SRS1041036\_contig\_number\_3173SRS1041090\_contig\_number\_7583SRS1041091\_contig\_number\_21168SRS1041095\_contig\_number\_5432SRS1041133\_contig\_number\_27098SRS1041134\_contig\_number\_4221SRS1041138\_contig\_number\_contig-100\_25606.25607SRS1041139\_contig\_number\_contig-100\_2606.41705SRS1041144\_contig\_number\_8749SRS1041146\_contig\_number\_contig-100\_2712.21191SRS104311\_contig\_number\_1555SRS104327\_contig\_number\_contig-100\_34.54302SRS104400\_contig\_number\_58883SRS104485\_contig\_number\_827SRS104975\_contig\_number\_contig-100\_2705.81772SRS105153\_contig\_number\_contig-100\_1369.230764SRS1054716\_contig\_number\_contig-100\_215.90469SRS1055049\_contig\_number\_10859SRS1055056\_contig\_number\_13221SRS142503\_contig\_number\_35311SRS142503\_contig\_number\_contig-100\_6198.142749SRS143085\_contig\_number\_contig-100\_161.49214SRS143342\_contig\_number\_19968SRS143895\_contig\_number\_contig-100\_293.263700SRS143991\_contig\_number\_contig-100\_1937.177253SRS144183\_contig\_number\_35408SRS144362\_contig\_number\_contig-100\_23112.23112SRS144537\_contig\_number\_contig-100\_552.250874SRS144714\_contig\_number\_5700SRS146764\_contig\_number\_24265SRS146812\_contig\_number\_contig-100\_3415.324807SRS147139\_contig\_number\_45534SRS147346\_contig\_number\_54894SRS147377\_contig\_number\_contig-100\_560.80581SRS147425\_contig\_number\_contig-100\_395.82425SRS147614\_contig\_number\_2236SRS147766\_contig\_number\_13225SRS148196\_contig\_number\_contig-100\_853.256123SRS148196\_contig\_number\_contig-100\_1667.256937SRS148253\_contig\_number\_580SRS148424\_contig\_number\_contig-100\_2476.198343SRS148721\_contig\_number\_42918SRS148817\_contig\_number\_14153SRS149244\_contig\_number\_8944SRS149784\_contig\_number\_23757SRS893252\_contig\_number\_19670SRS893259\_contig\_number\_contig-100\_828.54659SRS893288\_contig\_number\_contig-100\_57.43187SRS893295\_contig\_number\_contig-100\_656.83280SRS893300\_contig\_number\_contig-100\_2657.2658SRS893341\_contig\_number\_contig-100\_147.98051SRS893373\_contig\_number\_26326SRS893378\_contig\_number\_19102
species
39491
144

6
family
186806

6
genus
1730


SRS014683\_contig\_number\_19488SRS019397\_contig\_number\_11362SRS042284\_contig\_number\_7198SRS147272\_contig\_number\_2308SRS148319\_contig\_number\_contig-100\_3600.104415SRS893279\_contig\_number\_contig-100\_228.102283
species
39485
6
